# Multidrug Resistance Dynamics in Salmonella in Food Animals in the United States: An Analysis of Genomes from Public Databases

**DOI:** 10.1128/Spectrum.00495-21

**Published:** 2021-10-27

**Authors:** João Pires, Jana S. Huisman, Sebastian Bonhoeffer, Thomas P. Van Boeckel

**Affiliations:** a Institute for Environmental Decisions, ETH Zurich, Zurich, Switzerland; b Institute of Integrative Biology, ETH Zurich, Zurich, Switzerland; c Swiss Institute of Bioinformatics, Lausanne, Switzerland; d Center for Disease Dynamics, Economics & Policy, New Delhi, India; Dublin City University

**Keywords:** nontyphoidal *Salmonella*, antimicrobial resistance, food animals, genomic epidemiology, public data, *Salmonella*, surveillance

## Abstract

The number of bacterial genomes deposited each year in public databases is growing exponentially. However, efforts to use these genomes to track trends in antimicrobial resistance (AMR) have been limited thus far. We used 22,102 genomes from public databases to track AMR trends in nontyphoidal Salmonella in food animals in the United States. In 2018, genomes deposited in public databases carried genes conferring resistance, on average, to 2.08 antimicrobial classes in poultry, 1.74 in bovines, and 1.28 in swine. This represents a decline in AMR of over 70% compared to the levels in 2000 in bovines and swine, and an increase of 13% for poultry. Trends in resistance inferred from genomic data showed good agreement with U.S. phenotypic surveillance data (weighted mean absolute difference ± standard deviation, 5.86% ± 8.11%). In 2018, resistance to 3rd-generation cephalosporins in bovines, swine, and poultry decreased to 9.97% on average, whereas in quinolones and 4th-generation cephalosporins, resistance increased to 12.53% and 3.87%, respectively. This was concomitant with a decrease of *bla*_CMY-2_ but an increase in *bla*_CTX-M-65_ and *gyrA* D87Y (encoding a change of D to Y at position 87). Core genome single-nucleotide polymorphism (SNP) phylogenies show that resistance to these antimicrobial classes was predominantly associated with Salmonella enterica serovar Infantis and, to a lesser extent, S. enterica serovar Typhimurium and its monophasic variant I 4,[5],12:i:−, whereas quinolone resistance was also associated with S. enterica serovar Dublin. Between 2000 and 2018, trends in serovar prevalence showed a composition shift where *S.* Typhimurium decreased while *S.* Infantis increased. Our findings illustrate the growing potential of using genomes in public databases to track AMR in regions where sequencing capacities are currently expanding.

**IMPORTANCE** Next-generation sequencing has led to an exponential increase in the number of genomes deposited in public repositories. This growing volume of information presents opportunities to track the prevalence of genes conferring antimicrobial resistance (AMR), a growing threat to the health of humans and animals. Using 22,102 public genomes, we estimated that the prevalence of multidrug resistance (MDR) in the United States decreased in nontyphoidal Salmonella isolates recovered from bovines and swine between 2000 and 2018, whereas it increased in poultry. These trends are consistent with those detected by national surveillance systems that monitor resistance using phenotypic testing. However, using genomes, we identified that genes conferring resistance to critically important antimicrobials were associated with specific MDR serovars that could be the focus for future interventions. Our analysis illustrates the growing potential of public repositories to monitor AMR trends and shows that similar efforts could soon be carried out in other regions where genomic surveillance is increasing.

## INTRODUCTION

Nontyphoidal Salmonella is one of the most common agents of gastrointestinal disease globally (1). In the United States, nontyphoidal Salmonella is the second-most-frequent bacterium causing foodborne illness and the first bacterial pathogen in terms of hospitalizations and deaths (2). For severe infections, antimicrobial chemotherapy with 3rd-generation cephalosporins or fluoroquinolones is recommended (3). Therefore, antimicrobial resistance (AMR) in Salmonella is considered a serious public health threat (4).

Food animals are the principal reservoir for Salmonella, which is transmitted to humans through contaminated food products (5, 6). Reducing the burden of AMR in Salmonella in animals requires a combination of the following steps: (i) strong biosafety measures along the food-chain (7), (ii) ambitious antibiotic stewardship initiatives (8), and (iii) systematic monitoring of AMR levels to evaluate progress on the first two steps over time. In 1996, the National Antimicrobial Resistance Monitoring System (NARMS) was established to monitor AMR in humans and animals in the United States using phenotypic testing (9, 10). Since 2014, NARMS also includes a genomic surveillance component, albeit this is currently limited to a fraction of the isolates tested (9).

Meanwhile, between 2000 and 2018, a large number of epidemiological surveys on Salmonella that used next-generation sequencing (NGS) were conducted by independent research groups (11) in the United States. These studies helped in understanding the mechanisms of Salmonella host adaptation (12) and the emergence and spread of multidrug-resistant (MDR) clones (13) or investigated the origins of outbreaks (14). The multiplication of these surveys has led to an exponential increase in the number of genomes deposited in public databases (15), thereby allowing new opportunities for passive surveillance of Salmonella based on genetic traits.

In the United States, the abundance of passive genomic surveillance efforts for Salmonella, combined with the existence of a robust systematic surveillance system (NARMS), provides a unique opportunity to compare AMR trends inferred from genomes in public repositories with those obtained from phenotypic testing. In particular, this natural experiment could help calibrate the relationship between genomic and phenotypic resistance that could be used in other regions, including low-income regions, where genomic surveillance is being scaled up (16, 17).

The expansion of genomic surveillance programs enables the extraction of important information for designing public health measures. First, it allows the detection of acquired resistance genes or point mutations responsible for resistance phenotypes (both referred to hereinafter as ARGs), which is not feasible using antimicrobial susceptibility testing (18). Second, NGS enables the performance of *in silico* serovar detection, including uncommon serotypes that would otherwise only be typed in reference laboratories (19). The identification of ARGs of concern could be used to target epidemiological investigations and interventions to prevent their spread (20).

However, inferring temporal trends from genomes available in public databases comes with challenges. First, the quality (e.g., *N*_50_ and number of contigs) of deposited genomes may vary (21). Using quality-checked assemblies provided by several databases ensures data comparability across studies (21). Second, the metadata associated with the genomes in public databases are heterogenous (22), and these must be harmonized before defining inclusion criteria for epidemiological evaluations (22). Third, the number of genomes deposited by year is imbalanced, and representative temporal weighting must be applied to mitigate the impact of data-poor years while inferring trends in resistance.

In this study, we compiled a curated data set of nontyphoidal *Salmonella* genomes associated with food animals found in public databases. We report on trends in the prevalence of ARGs inferred from genomes for poultry, bovines, and swine in the United States and compare these with trends reported by NARMS.

## RESULTS

### Data set description.

We screened and harmonized the metadata of nontyphoidal Salmonella genome assemblies (hereinafter referred to as “genomes”) available in public databases containing a food animal host and a country of origin until 31 December 2018 (Fig. S1 and Table S1 in the supplemental material). We retrieved 21,689 genomes from EnteroBase (98.1%), 375 genomes from Pathosystems Resource Integration Center (PATRIC, 1.7%), and 38 genomes from the National Center for Biotechnology Information (NCBI, 0.17%). Most genomes were isolated in 2017 (*n* = 5,735, 22.8%) (Fig. S2). The majority of genomes were associated with poultry (*n* = 13,047, 59.03%), followed by bovines (*n* = 4,501, 20.36%), and swine (*n* = 3,874, 17.53%). The average lag times between genome isolation and release in public databases were 1.92, 1.45, and 1.19 years for bovines, poultry, and swine, respectively.

### Temporal trends in resistance.

We tracked the trends in MDR by analyzing the numbers of genes conferring resistance to different antimicrobial classes (see Materials and Methods and Tables S2 and S3) using a negative binomial model. Additionally, we weighted all observations by the U.S. food animal population (population correction unit [PCU]) and number of genomes per year per host (see Materials and Methods).

**FIG 1:**
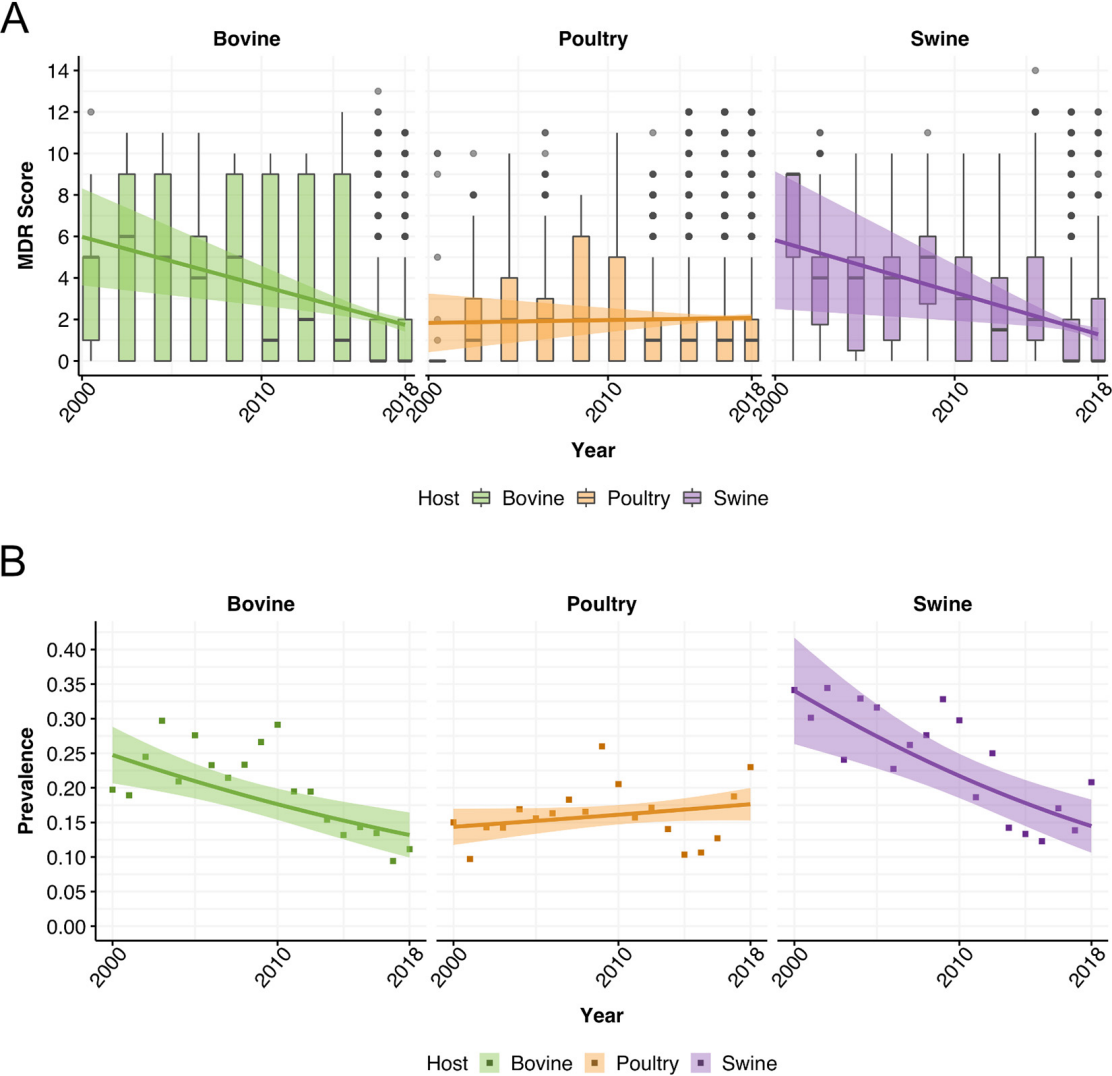
Temporal trends of multidrug resistance. (A) Temporal trends of the average numbers of antimicrobial classes Salmonella genomes in food animals are resistant to (multidrug resistance [MDR] scores). Solid lines represent the fitted MDR scores obtained from the negative binomial regression, and shading the 95% confidence intervals. (B) Temporal trends of the prevalences of isolates with resistance to ≥3 antimicrobials in NARMS. Solid lines represent the fitted prevalences from quasibinomial regressions, and shading the 95% confidence intervals.

Between 2000 and 2018, the average numbers of ARGs carried by genomes decreased in bovines and swine, but increased in poultry (Fig. 1A; Table S4). In 2000, genomes carried, on average, genes conferring resistance to 5.98 (95% confidence interval [CI], 3.64 to 8.32) antimicrobial classes in bovines, 5.82 (95% CI, 2.50 to 9.14) in swine, and 1.84 (95% CI, 0.42 to 3.25) in poultry. In 2018, resistance was more than halved in both bovines and swine: genomes carried, on average, genes conferring resistance to 1.74 (95% CI, 1.41 to 2.07) antimicrobial classes in bovines and 1.28 (95% CI, 0.96 to 1.60) in swine. Conversely, the MDR score for poultry increased to 2.08 (95% CI, 1.89 to 2.26). In NARMS surveillance data, resistance to three or more antimicrobial classes decreased over the same time period for bovines (from 24.7% to 13.2%) and swine (from 34.0% to 14.5%) but increased in poultry (from 14.4% to 17.6%) (Fig. 1B).

**FIG 2:**
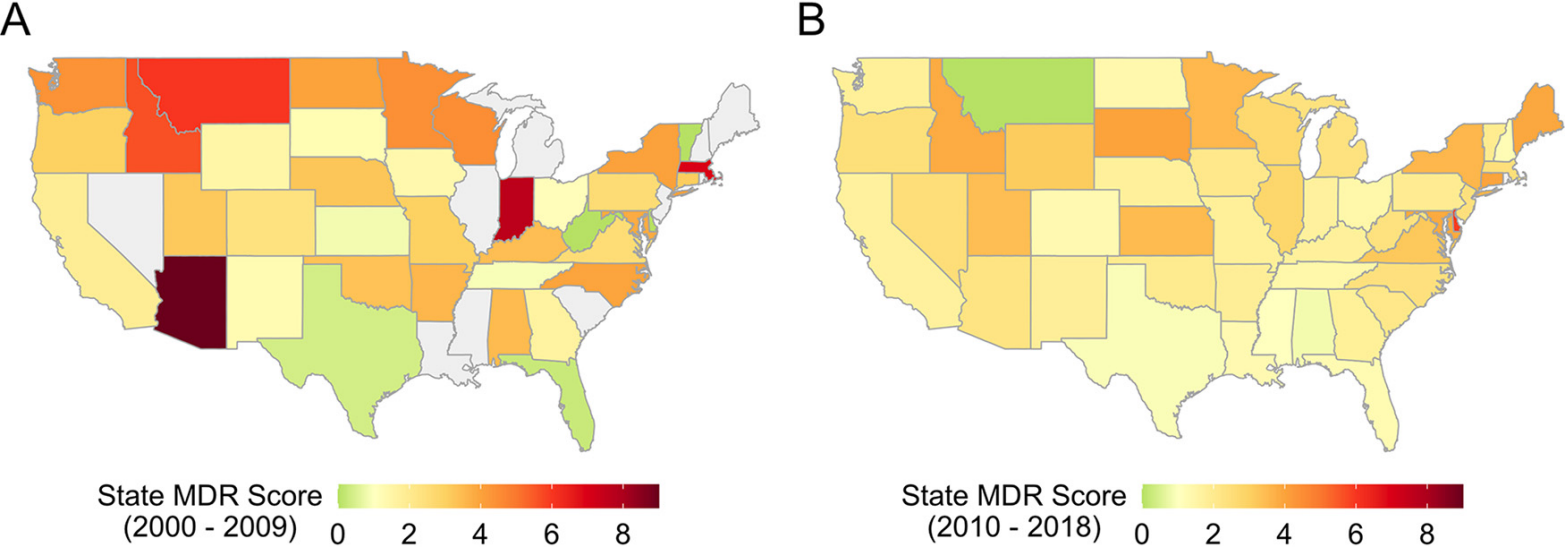
Multidrug resistance (MDR) scores by U.S. state. Gray states indicate no data were available. (A) Mean MDR scores in 2000 to 2009. (B) Mean MDR scores in 2010 to 2018.

The MDR score decreased across multiple states (Fig. 2; Fig. S3). In 2018, the highest MDR scores were found in the Midwest and Northwest (Fig. 2B).

### Resistance by antimicrobial class.

**FIG 3:**
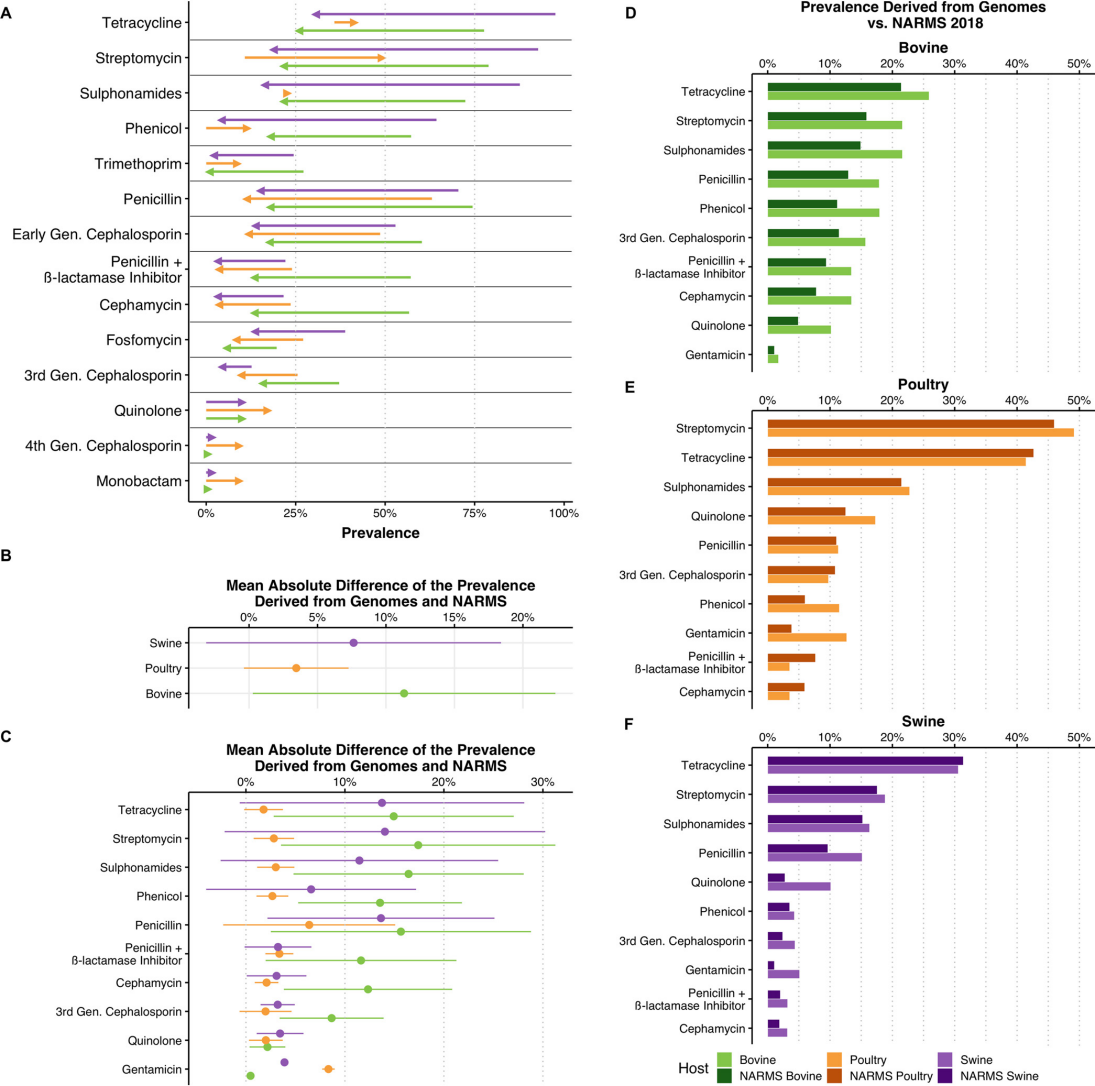
(A) Changes in prevalences of antimicrobial resistance inferred from genomes in the public domain between 2000 and 2018. Only significant temporal trends are shown (5% significance level). (B) Weighted mean absolute differences between the prevalences obtained from genomes and from NARMS for different hosts across all years and antimicrobial classes. (C) Weighted mean absolute differences between the prevalences obtained from genomes and from NARMS for individual antimicrobial classes across all years. (D to F) Comparison of the prevalences of resistance derived from genomes and from NARMS 2018 in bovines (D), poultry (E), and swine (F).

We inferred resistance prevalence trends for all antimicrobial classes based on ARGs’ predicted resistance phenotypes using a logistic regression. Fitted prevalence values for 2018 were compared to the phenotypic resistance prevalence for different antimicrobials available in NARMS (9). Between 2000 and 2018, decreasing trends were observed in 11 of 17 antimicrobials for both bovines and swine and in 6 of 17 in poultry (Fig. 3A; Table S5). Increases in resistance were only reported for quinolones, 4th-generation cephalosporins, and monobactams in all hosts. In poultry, increasing trends were additionally observed for phenicols, streptomycin, sulfonamides, and tetracyclines (Fig. 3A). Resistance to polymyxins and gentamicin was stable. Carbapenem resistance was not identified in genomes.

In poultry, resistance to streptomycin presented the highest prevalence recorded in 2018 (49.2%, 95% CI [46.3% to 52%]). A high prevalence of resistance was also observed for tetracyclines (41.4% [38.7% to 44.2%]) and, to a lesser extent, sulfonamides (22.8% [20.4% to 25.1%]) and quinolones (17.3% [14.9% to 19.7%]). All remaining antimicrobials had prevalences of resistance below 15%, including penicillins (11.3% [9.9% to 12.8%]) and 3rd-generation cephalosporins (9.8% [8.4% to 11.2%]). In poultry, the weighted mean absolute difference (MAD) between the prevalence derived from genomes and the prevalence reported in NARMS was 3.23% ± 3.94% across years and antimicrobial classes (Fig. 3D). Using genomes from public databases, we observed prevalence differences above 5% compared to the prevalence in NARMS in 2018 for gentamicin (12.7% versus 3.2% in NARMS) and phenicols (11.5% versus 3.2% in NARMS).

In 2018, the highest prevalences of resistance in swine were associated with tetracyclines (30.6% [25% to 36.1%]) and streptomycin (18.8% [14.7% to 23%]). Lower prevalences of resistance were observed for sulfonamides (16.3% [12.6% to 20.1%]), penicillins (15.2% [12.3% to 18%]), fosfomycin (13.6% [10.7% to 16.5%]), and quinolones (10.1% [6.8% to 13.5%]). All other antimicrobials had prevalences below 10%. Swine had a MAD between the prevalence derived from genomes and the prevalence reported by NARMS across years and antimicrobials of 7.42% ± 10.88% (Fig. 3E). In 2018, prevalence differences above 5% between those obtained from genomes and NARMS in swine were observed for penicillins (15.2% versus 10% in NARMS) and quinolones (10.1% versus 2.6% in NARMS).

In bovines, the prevalences of resistance to all antimicrobials were below 30% in 2018. The highest prevalences were observed for tetracyclines (25.9% [21.8% to 30%]), streptomycin (21.6% [17.8% to 25.4%]), sulfonamides (21.6% [17.8% to 25.4%]), and amphenicols (20.1% [16.5% to 21.1]). Lower prevalences were identified for penicillins (17.9% [14.7% to 21.1%]), 3rd-generation cephalosporins (15.7% [12.5% to 18.9%]), and penicillins in combination with β-lactamase inhibitors (13.4% [10.5% to 16.4%]). In bovines, the MAD between the prevalences derived from genomes and from NARMS across years and hosts was higher than those of the other hosts (11.59% ± 10.91%) (Fig. 3B). In bovines in 2018, differences of more than 5% in the prevalences obtained from genomes and from NARMS were observed for all antimicrobial classes (up to +7.93%).

**FIG 4:**
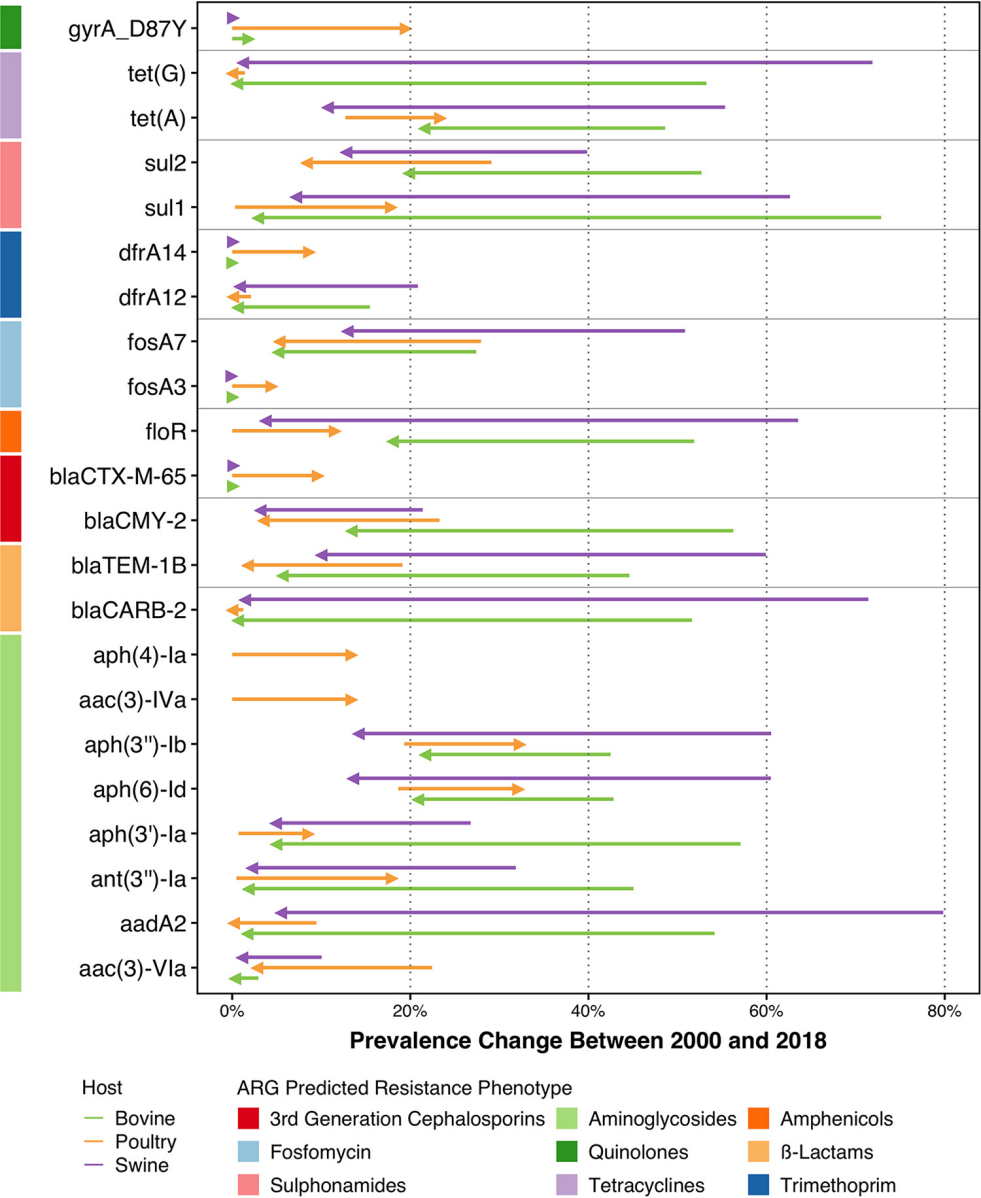
Changes in the prevalences of antimicrobial resistance genes between 2000 and 2018. Only significant temporal trends are shown (5% significance level). Colored arrows indicate the animal host, and bars represent the predicted phenotypes conferred by ARGs.

We also analyzed the temporal prevalences of individual ARGs using a logistic regression. The prevalences of genes conferring resistance to critically important antimicrobials declined between 2000 and 2018 (Table S6), including *bla*_CMY-2_ (3rd-generation-cephalosporin resistance) and *fosA7* (fosfomycin resistance). *bla*_CMY-2_ decreased from 56.3% to 13.4% in bovines, from 23.3% to 3.5% in poultry, and from 21.4% to 3.2% in swine. For *fosA7*, the prevalences decreased from 50.8% to 12.9% in swine, from 27.9% to 5.3% in poultry, and from 27.4% to 5.1% in bovines. Conversely, the 4th-generation-cephalosporin ARG *bla*_CTX-M-65_ increased the most in poultry, from 0.0007% to 9.7%. The prevalences of sulfonamide ARG *sul2*, penicillin ARGs *bla*_TEM-1_ and *bla*_CARB-2_, and aminoglycoside ARGs *aadA2* and *aac(*3*)-VIa* also declined in chicken, bovines, and pigs. For the chloramphenicol resistance gene *floR*, an increase was observed in poultry (from 0.004% to 11.6%), but decreases were observed for both bovines (from 51.9% to 18.0%) and swine (from 63.3% to 3.7%). These dynamics were also observed for the *tetA* gene, for which the prevalence increased in poultry (from 12.7% to 23.4%) and decreased in both bovines (from 48.6% to 21.6%) and swine (from 55.3% to 10.7%). Similarly, increasing dynamics in poultry and decreasing dynamics in bovines and swine were observed for several aminoglycoside ARGs, including *aph(3″)-Ib*, *aph(*6*)-Id*, *aph(3′)-Ia*, and *ant(3″)-Ia* (Fig. 4).

Increases in the prevalences of the following genes were only observed for poultry (Fig. 4; Table S6): aminoglycoside ARGs *aph(*4*)-Ia* (from 0.001%% to 13.5%) and *aac(*3*)-IVa* (from 0.01% to 13.5%).

For point mutations, the only significant increases in prevalence were associated with the *gyrA* mutation D87Y (encoding a change of D to Y at position 87), conferring resistance to quinolones and associated with poultry (from 0.0003% to 19.6%) (Fig. 4).

### Serovar prevalence and association with resistance.

**FIG 5:**
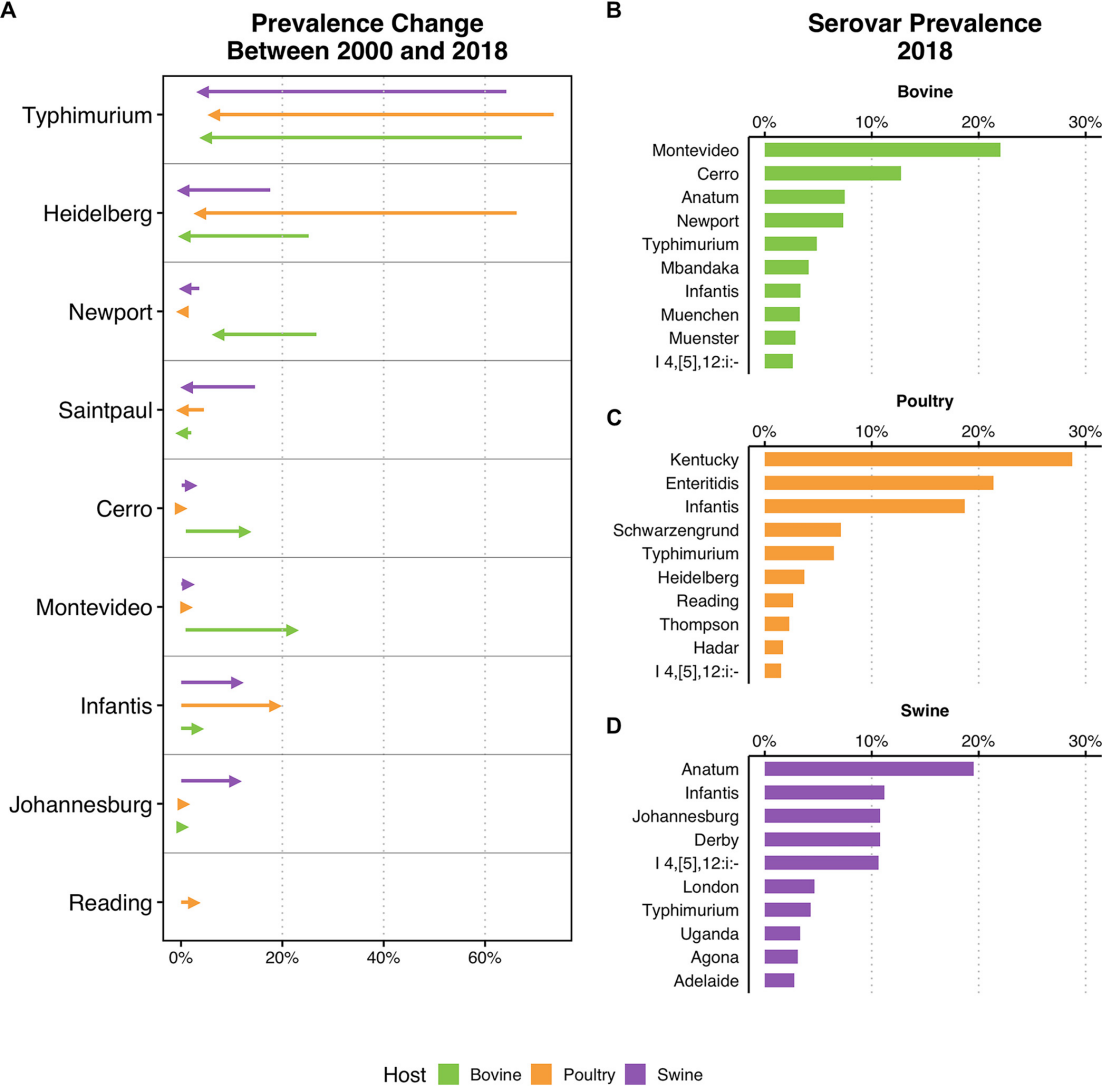
(A) Temporal trends for Salmonella serovar prevalences between 2000 and 2018. Only serovars with statistically significant temporal trends are shown (5% significance level). (B to D) Most frequent serovars in 2018 in bovine genomes (B), poultry genomes (C), and swine genomes (D).

We inferred serovar prevalences in different hosts for the 50 most frequent serovars in our data set using a logistic regression (Fig. 5; Table S7). Salmonella enterica serovar Typhimurium showed the greatest decreases in prevalence across all hosts between 2000 and 2018: from 73.5% to 6.5% in poultry, 67.2% to 4.9% in bovines, and from 64.2% to 4.3% in swine (Fig. 5A). Decreases were also observed in S. enterica serovar Heidelberg in poultry (from 66.2% to 3.7%), S. enterica serovar Newport in bovines (from 26.7% to 7.3%), and S. enterica serovar Saintpaul in swine (from 14.6% to 1.1%). Among serovars with decreasing prevalences, *S.* Typhimurium presented lower MDR scores from 2010 to 2018 than from 2000 to 2009 across all hosts (Fig. S4).

Prevalence increases were also observed for some serovars, including S. enterica serovar Infantis: from 0.06% to 18.7% in poultry, 0.03% to 11.2% in swine, and 0.009% to 3.3% in bovines. Increases were also observed for S. enterica serovar Cerro (from 1.0% to 12.7%) and S. enterica serovar Montevideo (from 0.9% to 22.0%) in bovines, and S. enterica serovar Johannesburg in swine (from 0.04% to 10.8%). In serovars displaying trends of increasing prevalence, only *S.* Infantis presented higher MDR scores in the 2010s than in the 2000s in poultry.

By 2018, *S.* Montevideo (22.4%), *S*. Cerro (12.7%), and S. enterica serovar Anatum (7.5%) were the most frequent serovars in bovine genomes (Fig. 5B; Table S8). S. enterica serovar Kentucky (28.7%), S. enterica serovar Enteritidis (21.4%), and *S*. Infantis (18.7%) were the most common in poultry, and serovars Anatum (19.5%), Infantis (11.2%), and Johannesburg (10.8%) in swine.

Positive correlations with resistance to critically important antimicrobials were observed with some serovars (Fig. S5 to S7). In bovines, positive correlations were observed for gentamicin resistance in *S.* Infantis, polymyxin resistance in S. enterica serovar Mbandaka, and quinolone resistance in S. enterica serovar Muenster (Fig. S5). In poultry, *S.* Infantis was associated with resistance to 4th-generation cephalosporins, monobactams, and gentamicin, *S*. Typhimurium was associated with resistance to 3rd-generation cephalosporins and cephamycins, and *S.* Heidelberg was associated with fosfomycin resistance (Fig. S6). Finally, in swine, the *S.* Typhimurium monophasic variant I 4,[5],12:i:− was associated with resistance to 4th-generation cephalosporins, monobactams, polymyxins, and quinolones, and both S. enterica serovar Agona and *S*. Infantis were associated with resistance to 3rd-generation cephalosporins and cephamycins (Fig. S7).

### Phylogenetic analysis.

**FIG 6:**
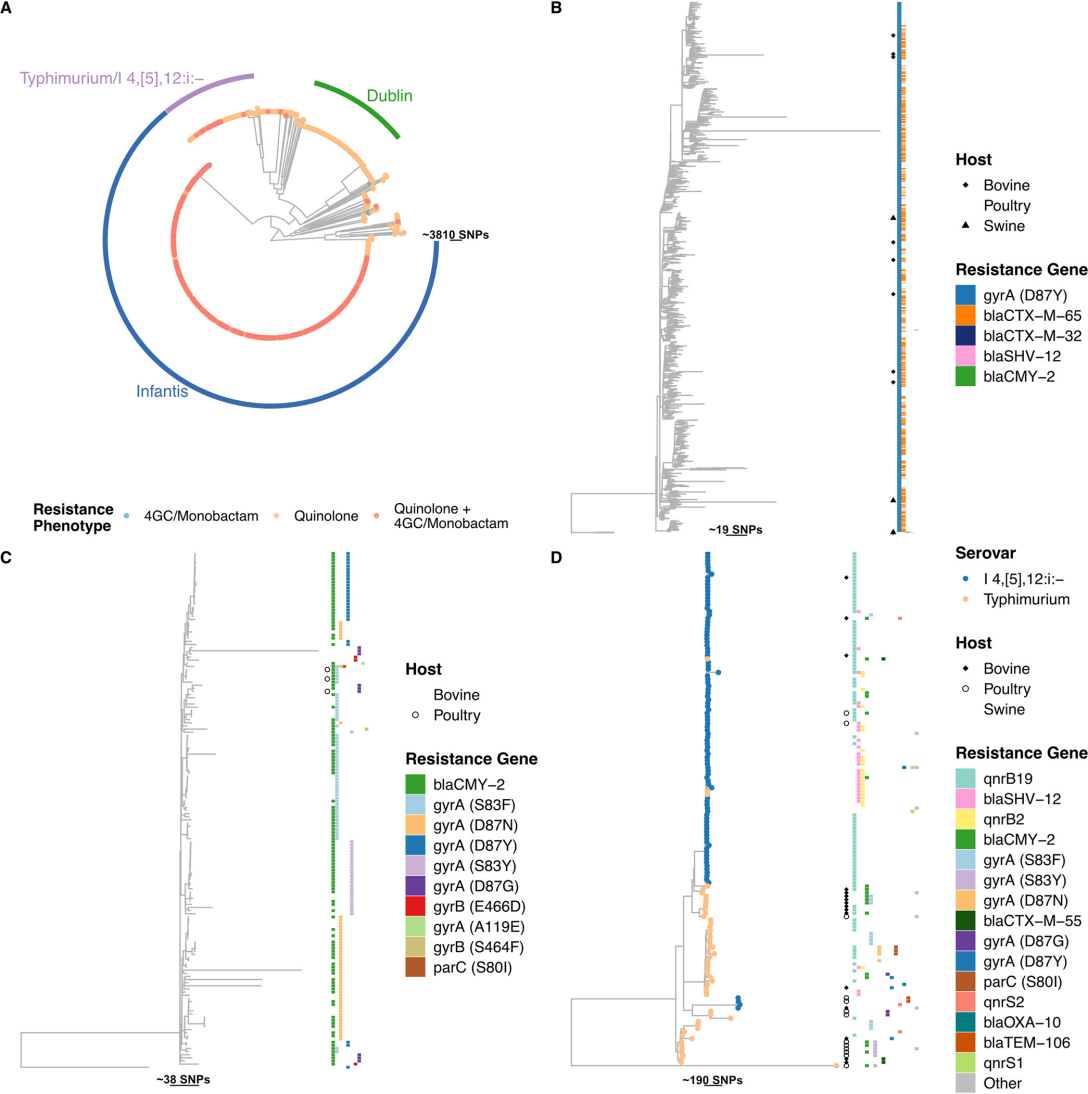
**(**A) Phylogenetic tree obtained from quinolone-, 4th-generation cephalosporin-, and monobactam-resistant Salmonella genomes (*n* = 1,643). 4GC, 4th-generation cephalosporin. (B) Subtree of Salmonella Infantis. Lack of a tip symbol indicates poultry origin. (C) Subtree of Salmonella Dublin. Lack of a tip symbol indicates bovine origin. (D) Subtree of Salmonella Typhimurium/I 4,[5],12:i:−. Lack of a tip symbol indicates swine origin.

We conducted a phylogenetic analysis using core genome single-nucleotide polymorphisms (SNPs) of 1,643 Salmonella genomes. We focused the analysis on genomes with predicted resistance against critically important antimicrobials that were increasing in prevalence (Fig. 6); the antimicrobials were quinolones, 4th-generation cephalosporins, and monobactams. In this subset, we identified 40 serovars, of which the majority (*n* = 1,372, 83.5%) were *S*. Infantis, S. enterica serovar Dublin, and *S*. Typhimurium and its monophasic variant I 4,[5],12:i:− (Fig. 6A).

Salmonella Infantis isolates comprised a large cluster (*n* = 1,055), the overwhelming majority of which were recovered from poultry (*n* = 1,043, 98.8%). The majority of genomes were quinolone resistant via the point mutation *gyrA* D87Y (*n* = 1,052, 99.8%). Of these, 54.5% (*n* = 575) cocarried *bla*_CTX-M-65_. These resistance genotypes were well mixed in the tree. Salmonella Dublin represented 165 (10%) of the isolates in the tree, of which 162 were recovered from bovines (98.2%). All isolates were quinolone resistant via point mutations, among which the *gyrA* mutations S83F (*n* = 51, 30.9%), S87N (*n* = 47, 28.5%), D87Y (*n* = 27, 16.4%), and S83Y (*n* = 25, 14.2%) were the most frequent. Genomes with the same mutations clustered together. In *S*. Dublin, no ARG conferring resistance to 4th-generation cephalosporins was identified. However, 122 (73.9%) genomes carried *bla*_CMY-2_ (3rd-generation cephalosporin resistance).

Finally, the *S*. Typhimurium/I 4,[5],12:i:− group included 152 genomes (9.3%), 123 (80.9%) of which were recovered from swine. The two serovars were separated into distinct branches of the tree. The gene *qnrB19* was the most frequent resistance mechanism detected in either serovar (66.7% and 30.2% in I 4,[5],12:i:− and Typhimurium, respectively). *S.* Typhimurium (*n* = 27, 50.9%) was more frequently associated with point mutations conferring resistance to quinolones than was serovar I 4,[5],12:i:− (*n* = 1, 1.01%). A small cluster of 23 serovar I 4,[5],12:i:− genomes carried *qnrB2* and *bla*_SHV-12_ (*n* = 14, 9.2%).

## DISCUSSION

To date, surveillance of AMR in Salmonella has relied on affordable and well established phenotypic assays (10). However, decreases in the costs of NGS technologies have helped generate an ever-growing body of genomes carrying resistance genes. We inferred trends in AMR from these genomes and made comparisons with the findings of an existing surveillance system that uses phenotypic testing in the United States (NARMS).

Between 2000 and 2018, the MDR scores calculated from genomes available in the public domain have decreased considerably in bovines and swine but increased in poultry (Fig. 1). These predictions are in line with those obtained from surveillance data in NARMS (9), although the magnitudes of the decreases in swine and bovines are smaller in data from NARMS (−57.4% in swine and −46.6% in bovines) than in data from genomes (−78% in swine and −70.9% in bovines). Nonetheless, our data suggest that for pigs and bovines, the decreases in Salmonella resistance were concomitant with reductions in the average numbers of ARGs per genome. This could not have been observed with phenotypic data alone. Since most isolates contain acquired resistance genes and not point mutations, this has the potentially important implication of a reduction in the probability of horizontal transfer of multiple ARGs to susceptible bacteria that could develop MDR phenotypes (23). In poultry, the slight increase in the MDR score is indicative of the opposite occurring.

We assessed the concordance between trends in AMR inferred in genomes in public databases and trends in AMR reported in NARMS. Across hosts, we observed an average divergence between the two data sources of 5.86% ± 8.11% (weighted mean absolute difference ± standard deviation across all hosts and antimicrobial classes) (Fig. 3). The smallest divergence was found for poultry genomes (3.45%). However, larger divergences were observed in swine (7.64%) and bovines (11.3%). The differences observed in β-lactam antimicrobials and gentamicin could be attributable to the fact that for resistance classification, NARMS uses clinical breakpoints rather than epidemiological cutoff values (ECOFFs) (3). However, this does not explain the divergences that were higher than 5% for other antimicrobial classes (e.g., tetracyclines and amphenicols), given that NARMS breakpoints and ECOFFs are identical (24). The divergences in bovines and swine could also be attributed to the lower volumes of data in these hosts and the data imbalance between the 2000 to 2009 period and the 2010 to 2018 period. In addition, this could explain the different magnitudes of the decreases in the MDR scores for these hosts. The increasing volume of genomic data expected to be uploaded in public databases in the future could help shed light on the nature of these divergences.

We observed that the most frequent ARGs in our data set correspond to those reported in the United States by NARMS (9). Across hosts, the temporal trends inferred from genomes available in the public domain showed decreases in the same ARGs [*sul2*, *bla*_TEM-1B_, *bla*_CMY-2_, *aadA2*, and *aac(*3*)-VIa*]. These genes have been associated with the same plasmids and with serotypes like Typhimurium, Heidelberg, and Newport (3, 25), all of which are predicted to be decreasing in our data (Fig. 5). Other ARGs displayed opposing trends across hosts, increasing in poultry and decreasing in swine and bovines, including *floR*, *sul1*, *tetA*, and several aminoglycoside ARGs (Fig. 4). Specific trends for poultry could indicate that different Salmonella isolates that are associated with different mobile genetic elements and ARGs circulate in this host. This is consistent with diverse serovar compositions observed for the different hosts (Fig. 5) and their different associations with resistance (Fig. S5 to S7) (3). Finally, using ARGs to track resistance trends also enabled the identification of succession dynamics in ARGs that confer resistance to the same antimicrobial (Fig. 4). This has identified the *fos* and *dfr* genes in all hosts, as well as the aminoglycoside ARGs in poultry (Fig. 4). This highlights the advantages of using NGS over antimicrobial susceptibility testing (17).

We attempted to identify lineages responsible for the increases in resistance to quinolones and 4th-generation cephalosporins (Fig. 3). From our phylogenies, we identified that the majority were *S.* Infantis isolates associated with poultry (Fig. 6A). Among these, 98% of *S.* Infantis isolates carried the *gyrA* D87Y mutation, and 50% also carried *bla*_CTX-M-65_. However, no specific cluster seemed to be responsible for the expansion of either resistance mechanism. The resistant *S.* Infantis isolates included in the phylogenetic analysis accounted for 63.4% of all Infantis isolates in our data set. However, the proportion of *S.* Infantis isolates resistant to quinolones or extended-spectrum β-lactams represented 86.2% of all isolates in 2018. The increase in *S.* Infantis isolates resistant to quinolones or extended-spectrum β-lactams is of concern given that these are antimicrobials used to treat severe infections caused by Salmonella (3). Furthermore, this serovar has been responsible for the majority of MDR isolates reported in the United States (26) and is associated with comparatively higher hospitalization rates in humans (27). Similarly, for *S.* Dublin and *S.* Typhimurium/I 4,[5],12:i:− (Fig. 6C and D), major sublineages were not identified in the tree, although some characteristics were clustered together, such as the different mutations in *S.* Dublin and the *qnrB2*+*bla*_SHV-12_ carriage in *S.* I 4,[5],12:i. The selected genomes of these serovars (resistant to quinolones or 4th-generation cephalosporins) only make up 26.1% of all Dublin and 13.4% of the I 4,[5],12:i:− genomes in the total data set. However, these serovars have been associated with MDR (28, 29), as well as with human infections in the United States (28). Therefore, close monitoring of these will be important to understand whether any of these sublineages will evolve to become dominant, especially considering that *S.* Dublin is well adapted to bovines and I 4,[5],12:i:− in swine (28, 29).

The phylogenies suggested that specific serovars were contributors to the emergence of MDR, particularly *S.* Infantis, although no specific sublineages were identified within serovars. This allows for the development of actions targeting specific serovars to prevent their spread. This could be achieved by administering vaccines against the serovars currently associated with higher probabilities of being MDR or carrying resistance to critically important antimicrobials, such as Infantis, I 4,[5],12:i:−, and Dublin (30–33). Moreover, as a majority of isolates are quinolone-resistant, designing rapid identification methods for serovars coupled with quinolone resistance determinants might identify areas where MDR serovars are highly prevalent and allow them to be rapidly controlled (34). This would reduce the spread of quinolone-resistant isolates and, collaterally, those resistant to extended-spectrum β-lactams, since nearly 47.2% of the isolates in the phylogenetic analysis contain ARGs to both quinolones and extended-spectrum β-lactams.

### Policy context.

The decrease in resistance over time reported in our analysis might be attributed to steps taken by U.S. agencies to combat AMR in food animals. This includes (i) the ban on extralabel use of fluoroquinolones in 1997 (35) and its complete ban in poultry in 2005 (36), (ii) the ban on extralabel use of 3rd-generation cephalosporins in 2012 (37), (iii) the promotion of judicious use of medically important antimicrobials in food animals by the Food and Drug Administration (FDA) (guidance number 209) (38), and (iv) changes in the regulations regarding the use of antimicrobials in feed in 2012 (and approved in 2015) that stipulate that antimicrobials can only be administered if prescribed by a veterinarian (guidance number 213) (39).

The restrictions in the use of quinolones could be the underlying reason why no acquired resistance genes against quinolones are predicted to be increasing in prevalence (only chromosomal point mutations). Similarly, the decrease of *bla*_CMY-2_ could be correlated with the 3rd-generation cephalosporin use restriction. However, the trends of increasing prevalences of quinolone and/or 4th-generation cephalosporin resistance are not explained by changes in antimicrobial use. Our analysis and other studies support that these changes are likely attributable to serovar compositional shifts, given the decreases in MDR serovars like Typhimurium and Heidelberg and the increase of Infantis (40). Explaining serovar trends remains challenging given the multiple factors that govern serovar lifestyle (e.g., host/environment adaptation, virulence factors, and antimicrobial resistance), which should be further explored (5, 6). Nonetheless, it has been suggested that the decrease in common serovars opens the opportunity for new serovars to emerge (41). The decrease in Typhimurium and Heidelberg has been associated with the widespread use of vaccines against these serovars (40). Therefore, we hypothesize that this opened the opportunity for Infantis to increase, in particular in poultry. Finally, we cannot discard the possibility of coselection, as these resistant serovars are also resistant to antimicrobials commonly used in food animals (5, 6).

Finally, U.S. agencies have focused their attention on postharvest actions to reduce the contamination of food products (41, 42), as well as tightening microbiological control within the hazard analysis and critical control points implemented by the Food Safety and Inspection Service (31, 43). This is because it is easier to control what will reach consumers at this stage (41). However, since Salmonella contamination (and consequently MDR Salmonella) in slaughterhouses is dependent on the colonization status of the animals on the farms (41), the next steps in reducing AMR in Salmonella could be reinforcing preharvest interventions (41).

### Limitations.

Our study comes with limitations. First, the genomes deposited in public databases do not result from systematic random sampling. Thus, the AMR trends presented here should be treated as event-based surveillance, which is not a substitute for systematic surveillance. Other works using event-based surveillance have helped to identify priorities for actions, after taking steps to harmonize data sources and accounting for potential reporting bias (44, 45). Second, to account for the lower abundance of data in the 2000 to 2009 period, we weighted all observations in our trend analysis to prevent overfitting to recent years. Third, correlation of our data with antimicrobial usage was not feasible at this stage since the FDA started reporting antimicrobial sales data only in 2009 (46). Fourth, we used assemblies in the bioinformatic analysis. While studies using raw reads have shown high accuracy in ARG detection, the gain is moderate (∼3%) compared to the accuracy of assembly-based approaches (47). In addition, using readily available assemblies has been recommended in the context of open pathogen surveillance (21). Fifth, low-level contamination might influence the detection of ARGs, the detection of point mutations, and phylogenetic analysis (48, 49). However, the vast majority of the genomes used in this study were obtained from EnteroBase (*n* = 21,689, 98.1%), which integrates a low-level contamination removal step in its assembly pipeline (50). Furthermore, during our bioinformatic analysis, we removed genomes that failed quality control modules using the SISTR and staramr tools. Although the detection of genes due to low-level contamination cannot be strictly ruled out, their influence on our analysis is likely limited because (i) just 2.2% (*n* = 1,069) of ARGs had a coverage or identity below 95%, (ii) in our analysis of the prevalences of resistance in individual classes, resistance was only counted once if multiple genes conferred resistance to the same antimicrobial class, and (iii) trends in ARGs were only assessed for those present in at least 1% of the genomes collected. Finally, for the phylogenetic analysis, we used a core genome alignment of gene clusters that are present in >99% of the genomes as input. This threshold has been previously suggested because it enables removing genes from the core genome that are associated with sequencing or assembly errors, thereby limiting the effects of low-level contamination on phylogenetic analyses (51).

### Future directions.

Finally, our results highlight the potential of ARGs to monitor resistance trends. In the near future, our approach could be adopted in other countries where sequencing efforts are currently expanding (17). In the United States, NARMS already collects genomes that can soon allow the use of ARGs to infer resistance trends (3, 9). In Europe, AMR surveillance in food animals using genomes should be achieved by 2025 (52). In low- and-middle income countries (LMICs) there are several financial and infrastructural barriers to NGS surveillance implementation (53). Nonetheless, several limitations can be overcome by international collaborations that facilitate capacity building. Several programs established by the U.S. Agency for International Development, the Wellcome Trust, and the Fleming Fund aim to provide support and capacity building in LMICs (16, 54). The value of such initiatives has recently been observed in the Philippines (55). Furthermore, other networks, such as the Global Microbial Identifier (56), PulseNet International (57), and GenomeTrakr (58), provide guidelines and external quality control to aid genome-based surveillance implementation.

## MATERIALS AND METHODS

### Harmonization of genomic data.

We screened three public databases for genome assemblies of Salmonella enterica deposited until 31 December 2018: the Nucleotide database of the National Center for Biotechnology Information (NCBI), EnteroBase (59), and Pathosystems Resource Integration Center (PATRIC) (Fig. S1) (60). Metadata was imported in R (version 3.6.0) and screened using text matching (see the supplemental material) (61). Metadata and accession numbers are shown in Table S1. Our analysis was focused on genomes from bovines, poultry, and swine.

### Bioinformatic analysis.

Multilocus sequence typing (MLST) and *in silico* serotyping were performed using the Salmonella *In Silico* Typing Resource (SISTR, version 1.1.1) (62). Sequence types and serovars for EnteroBase genomes were retrieved from metadata tables. SISTR outputs were used to focus our analysis on nontyphoidal Salmonella enterica subsp*. enterica.*

Acquired antimicrobial resistance genes were screened using ABRicate (version 1.0.1) (63) with the ResFinder (64) database (version of 22 April 2019). Antimicrobial resistance genes with an identity and coverage above 80% were kept. Predicted phenotypes were assigned based on the ResFinder database (see the supplemental material) (64). Salmonella-specific point mutations were screened using staramr (65) with the PointFinder database (version of 29 March 2021) (66). Mutations with at least 98% identity and 100% overlap were kept. Moreover, only mutations with known predicted phenotypes were kept for further analysis (*gyrA*, *gyrB*, and *parC* for quinolones and *pmrB* for colistin).

Finally, we checked the quality control modules that are performed as part of the SISTR and staramr pipelines. Assemblies that did not pass the quality control in either program were removed from further analysis (*n* = 21). Removal of low-level contamination reads and assembly quality control were performed as part of the EnteroBase internal pipeline.

### Statistical analysis of temporal trends.

We assessed temporal trends in resistance inferred from genomes in public databases and compared prevalence estimates with the data available in NARMS. First, we calculated a multidrug resistance (MDR) score for each isolate, i.e., the number of antimicrobial classes to which a genome is predicted to confer resistance (by carrying ARGs) (see the supplemental material, Tables S1 and S2). The temporal trends of the MDR scores were assessed using a negative binomial model with year and host as covariates (independent variables). Interactions between year and host were used to identify host-specific dynamics.

For assessing trends in prevalences of resistance for individual antimicrobial classes, we created a presence-absence matrix in which “1” represented the presence of a resistance phenotype and “0” was the absence. Each predicted resistance phenotype was counted once if multiple genes conferring resistance to the same antimicrobial class were present. We used a comparable approach for assessing trends in resistance in ARGs, in which only one ARG was counted in the presence-absence matrix if this gene was identified multiple times in the same genome. For ARGs, only those present in at least 1% of the genomes were used for the prevalence analysis, to ensure sufficient sample size. Based on NARMS surveillance reports, we identified host-specific trends using interactions between year and host for the prevalences of resistance and individual ARGs conferring resistance to tetracyclines, streptomycin, sulfonamides, phenicols, and trimethoprim. For the prevalences of individual serovars, we created a presence-absence matrix for the 50 most common serovars present in our data set. All models were weighted using the animal population correction unit (PCU) and the number of genomes available per year (see the supplemental material) and calibrated using a 10-fold cross-validation (67).

For NARMS data, we extracted prevalence values reported in the NARMS Now website. We performed MDR temporal trend analysis using the prevalences of resistance to three or more antimicrobial classes. We also analyzed the temporal trends of resistance to individual antimicrobial classes. In both cases, we analyzed data using a quasibinomial model with year and host as covariates. Observations were weighted by the PCU and the number of isolates that underwent antimicrobial susceptibility testing in NARMS per year. Finally, we compared the prevalence estimates obtained from NARMS and from genomes using the weighted mean absolute difference (MAD) for each antimicrobial class (*A*) across years using the following formula:

$${\text{MAD}}_{A}=\;\frac{\sum_{i=1}^{n}\left(\left|P_{\text{Genomes,}i}\;-\;P_{\text{NARMS,}i}\right|\times w_{i}\right)}{\sum_{i=1}^{n}w_{i}}$$

in which *i* is the year, *P*_Genomes,_*_i_* is the prevalence obtained for year *i* from genomes in the public domain, *P*_NARMS,_*_i_* is the prevalence obtained for year *i* from NARMS, and *w_i_* represents the number of genomes reported for year *i*.

### Association of serovar and resistance.

We compared the MDR scores for the periods of 2000 to 2009 and 2010 to 2018 for the most frequent serovars using a Wilcoxon rank sum test. For statistically significant χ^2^ tests, we proceeded with a *post hoc* analysis to identify specific serotype-phenotype associations by comparing the standardized residuals against the χ^2^ cumulative distribution. *P* values were adjusted using Bonferroni correction. Adjusted *P* values below 0.05 were considered statistically significant.

### Phylogenetic analysis of Salmonella isolates resistant to critically important antimicrobials with increasing prevalence trends.

We conducted a phylogenetic analysis to identify whether certain Salmonella lineages could be associated with the increasing trends of resistance to critically important antimicrobials (see “Statistical analysis of temporal trends,” above) (Fig. 3). We extracted the genomes with predicted resistance to quinolones, 4th-generation cephalosporins, and monobactams (*n* = 1,643). All assemblies were annotated with Prokka (version 1.13) (68), and a core genome alignment was obtained using Roary (version 3.13.0) (52). Single-nucleotide polymorphisms (SNPs) in the core genome were retrieved using SNP sites (version 2.5.1) (68), and the resulting output was used to build a phylogenetic tree using RAxML (version 8.2.4) (69) with 100 bootstraps. The phylogenetic tree was imported into R and annotated with ggtree (70).

### Data availability.

Assemblies used in this study are available in public databases. All identifiers for traceability, including database of origin, database internal identification number, and BioSample accession number (when applicable), are available in Table S1. Code used in this study and Table S2 can be found in the Zenodo repository with the following link: https://doi.org/10.5281/zenodo.5519129 (71).
