# Supplementary material for: Multidrug Resistance Dynamics in Salmonella in Food Animals in the United States: An Analysis of Genomes from Public Databases
Source: Microbiol Spectr. 2021 Oct 27;9(2):e00495-21. doi: 10.1128/Spectrum.00495-21 (PMC8549754; doi:10.1128/Spectrum.00495-21)
Supplement: SUPPLEMENTAL FILE 1 — Supplemental material. Download SPECTRUM00495-21_Supp_1_seq10.pdf, PDF file, 0.7 MB [file spectrum00495-21_supp_1_seq10.pdf]

Appendix for:

Multidrug Resistance Dynamics in *Salmonella* in Food-Animals in the United States: An Analysis of Genomes from Public Databases

João Pires[1*], Jana S. Huisman[2,3], Sebastian Bonhoeffer[2], and Thomas P. Van Boeckel[1,4]

Affiliations:

1. Institute for Environmental Decisions, ETH Zurich, Zurich, Switzerland
2. Institute of Integrative Biology, ETH Zurich, Zurich, Switzerland
3. Swiss Institute of Bioinformatics, Lausanne, Switzerland
4. Center for Disease Dynamics, Economics & Policy, New Delhi, India

This appendix contains:

I. Supplementary Materials and Methods
II. Appendix Figures
III. Legends for Appendix Tables
IV. Legend for Appendix Dataset

I. Supplementary Materials and Methods

1. Data Retrieval and Harmonization

1.1. Genomic Metadata Retrieval

We searched for all available *Salmonella enterica* assemblies recovered from food-animals released until the end of 2018 in three public genomic data repositories: the National Center for Biotechnology Information (NCBI) Nucleotide database(1), EnteroBase(2) and Pathosystems Resource Integration Center (PATRIC)(3). For NCBI Nucleotide, we queried Entrez Programming Utilities using the taxonomic identification of *S. enterica* ("taxid28901")(4). Resulting accession numbers were retrieved and used to retrieve associated metadata. For both EnteroBase and PATRIC, the entire metadata tables were downloaded.

1.2. Metadata Standardization

Metadata tables were imported into R (version 3.6.0)(5). Manipulation of metadata was performed with the tidyverse(6) (version 1.3.0), data.table(7) (version 1.12.8) and plyr(8) (version 1.8.6) packages.

All entries that did not report a country of origin or geographic coordinates were removed. Thereafter, we inspected isolation sources and to identify food-animal key words that allowed to reduce the datasets, but would maximize the number of hits. The resulting filtered datasets were then manually curated to exclude entries that did not meet the criteria of food-animal. We considered four levels of data aggregation for host attribution:

- **Source Niche:** highest level of aggregation and indicates whether samples were recovered from food-products (Food) or from the animals themselves (Poultry or Livestock);
- **Generic Host:** aggregation within animal-production group such as poultry, swine, bovine, ovine and caprine. The categories dairy, meat and environment were also introduced when no specific animal was given. The category environment denotes food-animal-related samples not collected directly from the animal or their food-products such as drag swabs, poultry litter, eggshells, animal bedding and barns;
- **Source Type:** indicates the specific animal from which the samples were collected;
- **Source Details:** contains the original sample description as input by the submitter.

Geographic coordinates were retrieved from metadata tables when available. Coordinates expressed in cardinal directions were converted to decimal degree. For entries without coordinates, an addresse was constructed based on the available information of the isolation location (country, province, state, region, city, zip code, etc.). We queried addresses for their decimal degree coordinates with the geocode function of the ggmap package(9) (version 3.0.0). Assemblies returning no coordinates were inspected manually and queried in Google Earth Pro(10). A column with country's three-letter code based on the ISO 3166-1 guidelines was also assigned.

Isolation dates were harmonized according to the ISO 8601 format (year-month-day) using lubridate(11) and anytime(12) packages. A dedicated column for year of isolation was also created. Finally, the NCBI BioSample and BioProject (when available) were also kept.

1.3. Creation of a Consensus Dataset and Assembly Download

We used the BioSample identifier to compare entries across databases and created a consensus dataset by removing duplicate entries. We primarily kept entries from EnteroBase given the dedicated pipeline this database has towards short-read sequences assembly, quality control, and molecular typing). Then we retrieved data from PATRIC and finally from NCBI RefSeq(13). EnteroBase derived assemblies were kindly provided by the curators, PATRIC assemblies were downloaded through the PATRIC Command Line Interface(14), NCBI assemblies were downloaded from the RefSeq database(13). Each entry has the original identifier from the database and a column indicating from which database it retrieved from.

2. Curation of Predicted Phenotypes

We extracted the predicted phenotypes from ResFinder database (https://bitbucket.org/genomicepidemiology/resfinder_db/src/master/, accessed 27th May 2020). We retrieved the predicted antibiotic family (Antibiotic Class) and specific antibiotics (Phenotype) to which they confer resistance. All antimicrobial resistance genes (ARGs) found in our dataset can be found in Supplementary Table 2. Phenotypes of genes with unassigned predicted phenotypes were inputted based on the closest match sequence match. In brief, we retrieved the sequence of such ARGs based on the available NCBI accession number and used Basic Local Alignment Search Tool (BLAST)(15) against the CARD database. Predicted phenotypes were assigned based on the gene with the best alignment score, but with a minimum of 97% identity. When no matches were found in CARD, we used NCBI's BLAST(16) instead. For the matches with the highest identity and coverage, we inspected the referred

manuscripts where such ARG and their respective resistance phenotypes were described. Finally, for ß-lactamase genes, we added cephalothin manually to the Phenotype column. This is because early generation cephalosporins were not included in the ResFinder phenotype list, although TEM-types(17), AmpC(18), OXA-Types(19) hydrolyze these ß-lactams. We removed *aac(6')-Iaa* from the dataset as this gene has been described as intrinsic to *S. enterica* and does not cause phenotypic resistance(20, 21).

In the case of point mutations, we only kept those that have known resistance phenotype in the PointFinder database (22), which can extracted directly from the staramr output(23).

3. Multidrug Resistance Score Calculation

We devised a metric to summarize multidrug resistance based on the number of different classes of antimicrobials an isolate is predicted to have resistance based on its content in ARGs (acquired ARGs or point mutations). We call this metric the Multidrug Resistance Score (MDR Score). We based this metric on microbiological resistance. In brief, microbiological resistance is identified when the minimal inhibitory concentration (MIC) is above the epidemiological cut-off (ECOFF) value(24) – the highest MIC for organisms devoid of phenotypically detectable acquired resistance mechanisms(25). We assume that all identified ARGs are functional and thus resulting MICs would be above the ECOFF. For the majority of ARGs, the phenotype would not be affected. However, it could affect the predicted phenotype ß-lactamase genes since for some variants the amino acid changes result in different resistance phenotypes(26). Although only 0.44% of the ß-lactamase genes had a coverage and identity below 100% in our study.

To calculate the MDR Score, we used a list of antimicrobials of clinical importance *Enterobacteriaceae* in relation to acquired resistance(27). We used cephalothin as a surrogate for cefazolin since they are both early generation cephalosporins.

The MDR score was computed as follow:

- For each genome, the unique predicted resistance phenotypes were identified, and antibiotics were grouped into the different molecular classes:
    - Aminoglycosides
    - Penicillins
    - Early Generation Cephalosporins
    - Cephamycins
    - 3rd Generation Cephalosporins
    - 4th Generation Cephalosporins
    - Monobactams
    - Carbapenems
    - Penicillins in combination with β-lactamase inhibitors
    - Quinolones
    - Trimethoprim
    - Sulphonamides
    - Phenicols
    - Tetracyclines
    - Polymyxins
    - Fosfomycin

- The MDR Score will increase by one when an antibiotic is assigned to one of the described molecular classes. If more ARGs confer resistance to the same molecular class, the MDR score still only increases by one.
- All genomes for which no ARGs are identified are assigned a MDR score of zero.

4. Final Dataset

The final dataset comprises 22,102 assemblies that belong to non-Typhoidal *Salmonella*. The final metadata table contains the following:

- Assembly ID: name of assembly ID as identified in the database;
- Database: name of repository from which said assembly was recovered;
- Collection Date: isolation date;
- Year: isolation year;
- ISO3: 3 letter code of the country of isolation;
- Latitude and Longitude: coordinates in decimal degree;
- Serovar: *Salmonella*'s Serovar;
- ST: *Salmonella*'s sequence type;
- BioSample: NCBI BioSample accession number;
- BioProject: NCBI BioProject accession number;
- Acquired Resistance: whether this assembly was found to contain ARGs or not;
- MDR Score: calculated MDR Score;

5. Model Weights Calculation

For the temporal trend analysis of resistance, we need to weight the observations relative to their representativeness in our dataset. To achieve this, we weighted all observations by the countries' Population Correction Unit (PCU) for each host (expressed as proportion) and corresponding isolation year times the proportion of genomes contributed by a given a country for a given year. We calculated PCU as described by Tiseo and colleagues (28) for all countries as follows:

$$PCU_{k,s} = An_{k,s}.\left(1 + \ n_{k,s}\right).\left(\frac{Y_k}{R_{\frac{CW}{LW},k}}\right)$$

where $An_{k,s}$ is the number of animal type, *k*, for each production system, *s* (intensive or extensive), in each country; $n_{k,s}$ is the number of production cycles for each animal type in each production system; Y_k is the quantity of meat in each country for each animal type; and $R_{CW/LW,k}$ is the carcass weight to live weight ratio for each animal type. The PCU allows for direct comparisons of animals raised for food in across countries. For some countries, PCU data was unavailable before 1999 for Belgium, before 1991 for Belarus, before 1992 for Czech Republic and Slovakia before, and before 1991 for Belarus, Croatia, Estonia, and Lithuania. In addition, no PCU data existed prior to 1985. In such cases, we assigned the PCU value corresponding to the earliest available year in the time series. PCU data can be found in Supplementary Dataset S1.

6. References

1. Home - Nucleotide - NCBI.

2. Enterobase.

3. PATRIC.

4. Taxonomy - NCBI.

5. R Core Team (2019),. R: The R Project for Statistical Computing.

6. Wickham H, Averick M, Bryan J, Chang W, McGowan L, François R, Grolemund G, Hayes A, Henry L, Hester J, Kuhn M, Pedersen T, Miller E, Bache S, Müller K, Ooms J, Robinson D, Seidel D, Spinu V, Takahashi K, Vaughan D, Wilke C, Woo K, Yutani H. 2019. Welcome to the Tidyverse. J Open Source Softw 4:1686.

7. Dowle M, Srinivasan A, Gorecki J, Chirico M, Stetsenko P, Short T, Lianoglou S, Antonyan E, Bonsch M, Parsonage H, Ritchie S, Ren K, Tan X, Saporta R, Seiskari O, Dong X, Lang M, Iwasaki W, Wenchel S, Broman K, Schmidt T, Arenburg D, Smith E, Cocquemas F, Gomez M, Chataignon P, Groves D, Possenriede D, Parages F, Toth D, Yaramaz-David M, Perumal A, Sams J, Morgan M, Quinn M, @javrucebo, @marc-outins, Storey R, Saraswat M, Jacob M, Schubmehl M, Vaughan D. 2019. data.table: Extension of "data.frame."

8. Wickham H. 2019. plyr: Tools for Splitting, Applying and Combining Data.

9. Kahle D, Wickham H. 2013. ggmap: Spatial Visualization with ggplot2. R J 5:144.

10. Google Earth.

11. Spinu V, Grolemund G, Wickham H, Lyttle I, Costigan I, Law J, Mitarotonda D, Larmarange J, Boiser J, Lee CH. 2020. lubridate: Make Dealing with Dates a Little Easier.

12. Eddelbuettel D. 2020. anytime: Anything to “POSIXct” or “Date” Converter.

13. O’Leary NA, Wright MW, Brister JR, Ciufo S, Haddad D, McVeigh R, Rajput B, Robbertse B, Smith-White B, Ako-Adjei D, Astashyn A, Badretdin A, Bao Y, Blinkova O, Brover V, Chetvernin V, Choi J, Cox E, Ermolaeva O, Farrell CM, Goldfarb T, Gupta T, Haft D, Hatcher E, Hlavina W, Joardar VS, Kodali VK, Li W, Maglott D, Masterson P, McGarvey KM, Murphy MR, O’Neill K, Pujar S, Rangwala SH, Rausch D, Riddick LD, Schoch C, Shkeda A, Storz SS, Sun H, Thibaud-Nissen F, Tolstoy I, Tully RE, Vatsan AR, Wallin C, Webb D, Wu W, Landrum MJ, Kimchi A, Tatusova T, DiCuccio M, Kitts P, Murphy TD, Pruitt KD. 2016. Reference sequence (RefSeq) database at NCBI: current status, taxonomic expansion, and functional annotation. Nucleic Acids Res 44:D733–D745.

14. GitHub - PATRIC3/PATRIC-distribution: For distributing patric related code.

15. BLAST - Basic Local Alignment Search Tool. The Comprehensive Antibiotic Resistance Database.

16. BLAST: Basic Local Alignment Search Tool.

17. Bonomo RA. 2017. β-Lactamases: A Focus on Current Challenges. Cold Spring Harb Perspect Med 7.

18. Jacoby GA. 2009. AmpC β-Lactamases. Clin Microbiol Rev 22:161.

19. Evans BA, Amyes SGB. 2014. OXA β-Lactamases. Clin Microbiol Rev 27:241.

20. Salipante SJ, Hall BG. 2003. Determining the limits of the evolutionary potential of an antibiotic resistance gene. Mol Biol Evol 20:653–659.

21. Neuert S, Nair S, Day MR, Doumith M, Ashton PM, Mellor KC, Jenkins C, Hopkins KL, Woodford N, de Pinna E, Godbole G, Dallman TJ. 2018. Prediction of Phenotypic Antimicrobial Resistance Profiles From Whole Genome Sequences of Non-typhoidal *Salmonella enterica*. Front Microbiol 9.

22. Zankari E, Allesoe R, Joensen KG, Cavaco LM, Lund O, Aarestrup FM. 2017. PointFinder: a novel web tool for WGS-based detection of antimicrobial resistance associated with chromosomal point mutations in bacterial pathogens. 10. J Antimicrob Chemother 72:2764–2768.

23. 2021. staramr. Python, National Microbiology Laboratory.

24. Ellington MJ, Ekelund O, Aarestrup FM, Canton R, Doumith M, Giske C, Grundman H, Hasman H, Holden MTG, Hopkins KL, Iredell J, Kahlmeter G, Köser CU, MacGowan A, Mevius D, Mulvey M, Naas T, Peto T, Rolain J-M, Samuelsen Ø, Woodford N. 2017. The role of whole genome sequencing in antimicrobial susceptibility testing of bacteria: report from the EUCAST Subcommittee. Clin Microbiol Infect 23:2–22.

25. Kahlmeter G. 2015. The 2014 Garrod Lecture: EUCAST – are we heading towards international agreement? J Antimicrob Chemother 70:2427–2439.

26. Bush K. 2018. Past and Present Perspectives on β-Lactamases. 10. Antimicrob Agents Chemother 62:e01076-18.

27. Magiorakos A-P, Srinivasan A, Carey RB, Carmeli Y, Falagas ME, Giske CG, Harbarth S, Hindler JF, Kahlmeter G, Olsson-Liljequist B, Paterson DL, Rice LB, Stelling J, Struelens MJ, Vatopoulos A, Weber JT, Monnet DL. 2012. Multidrug-resistant, extensively drug-resistant and pandrug-resistant bacteria: an international expert proposal for interim standard definitions for acquired resistance. Clin Microbiol Infect Off Publ Eur Soc Clin Microbiol Infect Dis 18:268–281.

28. Tiseo K, Huber L, Gilbert M, Robinson TP, Van Boeckel TP. 2020. Global Trends in Antimicrobial Use in Food Animals from 2017 to 2030. 12. Antibiotics 9:918.

29. Kassambara A. 2021. rstatix: Pipe-Friendly Framework for Basic Statistical Tests.

30. Ebbert D. 2019. chisq.posthoc.test: A Post Hoc Analysis for Pearson's Chi-Squared Test for Count Data.

II. Appendix Figures

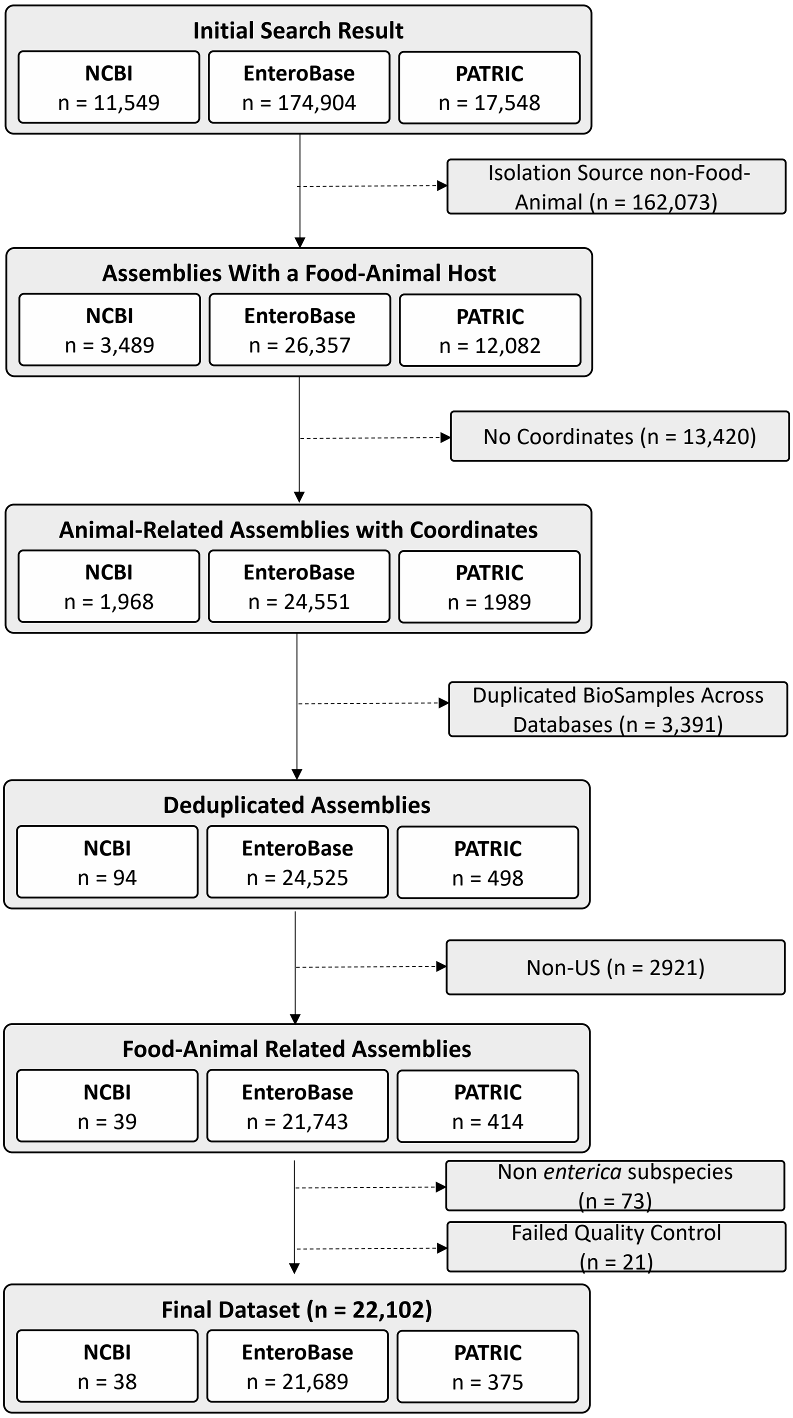

Figure S1. Number of genomes identified in public repositories and number of genomes excluded throughout the curation process. NCBI - National Center for Biotechnology Information (NCBI) Nucleotide database; PATRIC - Pathosystems Resource Integration Center.

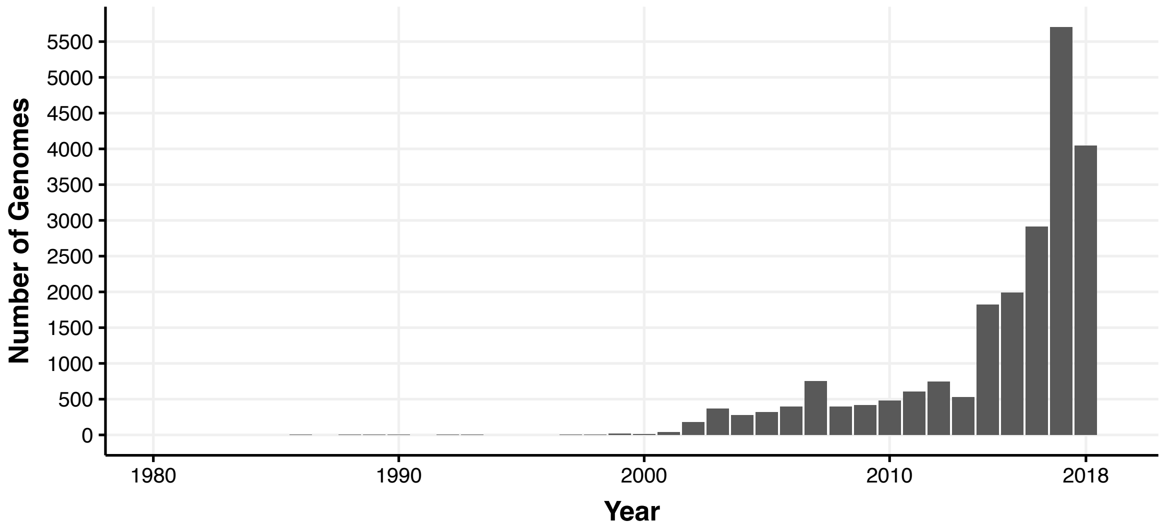

Figure S2. Distribution of the number of genomes per year.

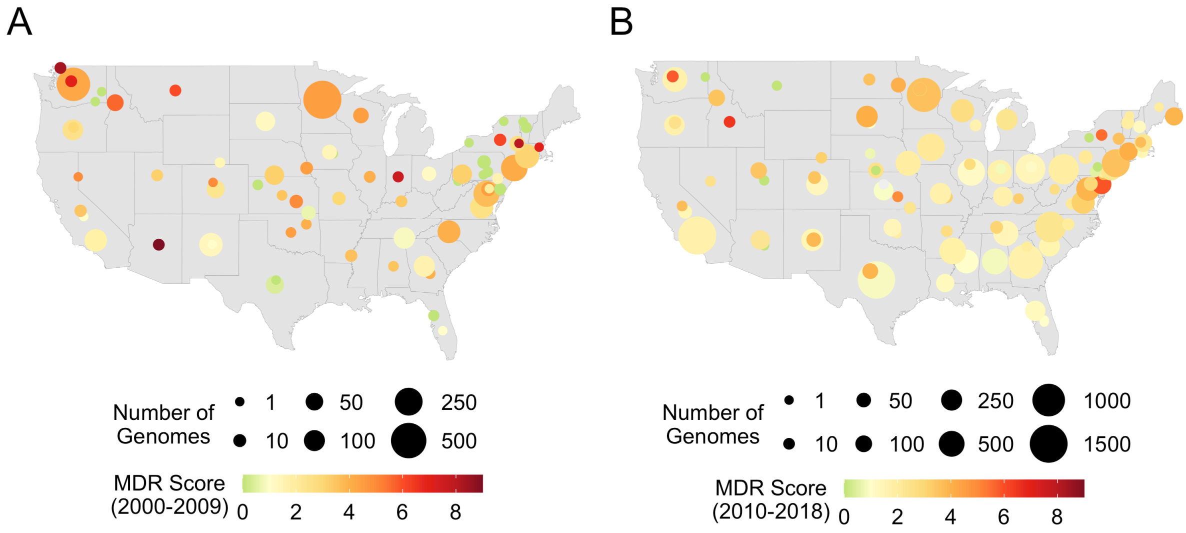

Figure S3. Distribution of the of Multidrug Resistance Score (MDR Score) across the United States. The dot size represents the number of genomes available for a single geographic coordinate. **A.** Distribution of the MDR Score between 2000-2009; **B.** Distribution of the MDR Score between 2010-2018.

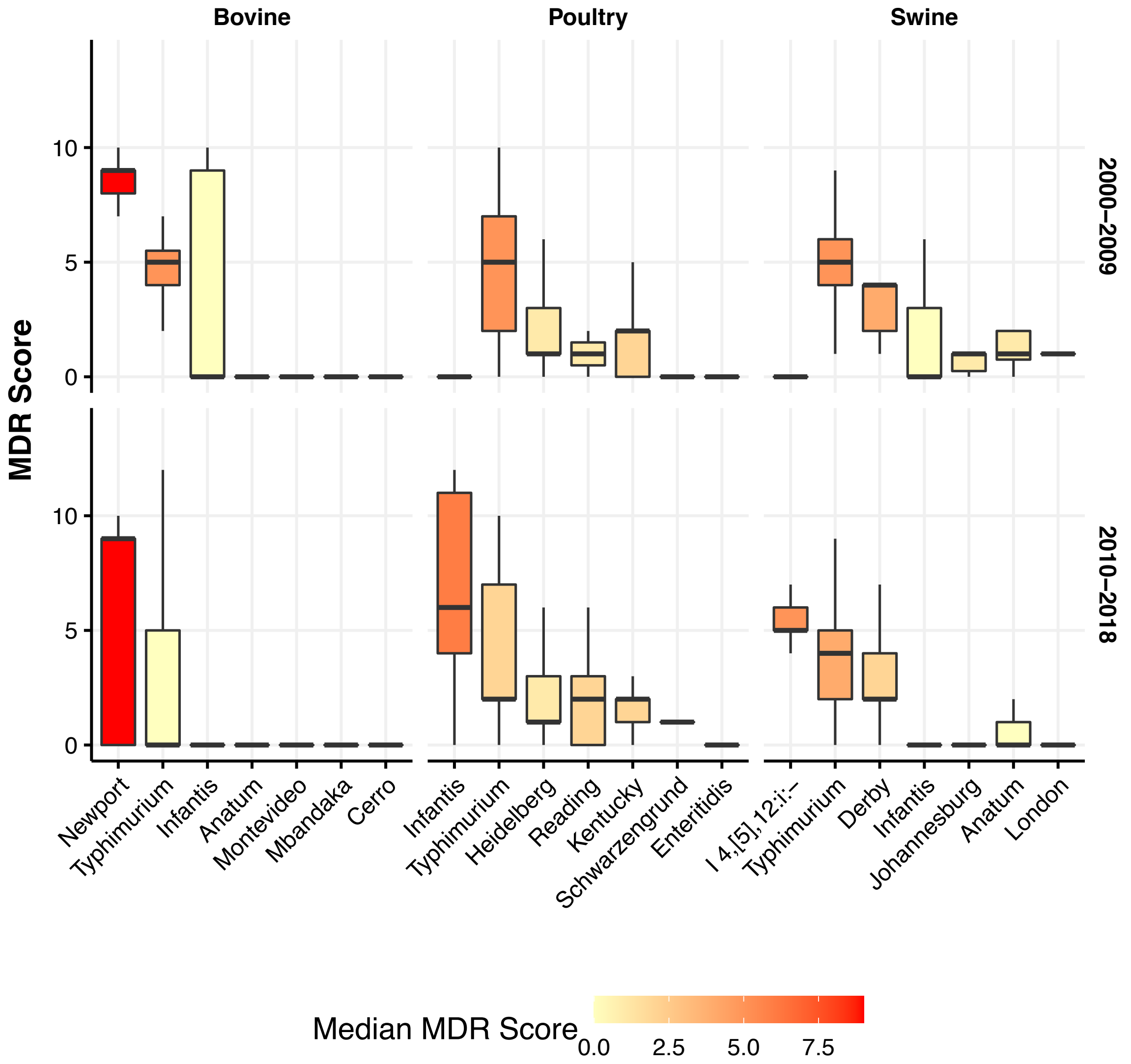

Figure S4. Distribution of the Multi Drug Resistance Score (MDR Score) per host per serovar in 2000s (2000-2009) and 2010s (2010-2018).

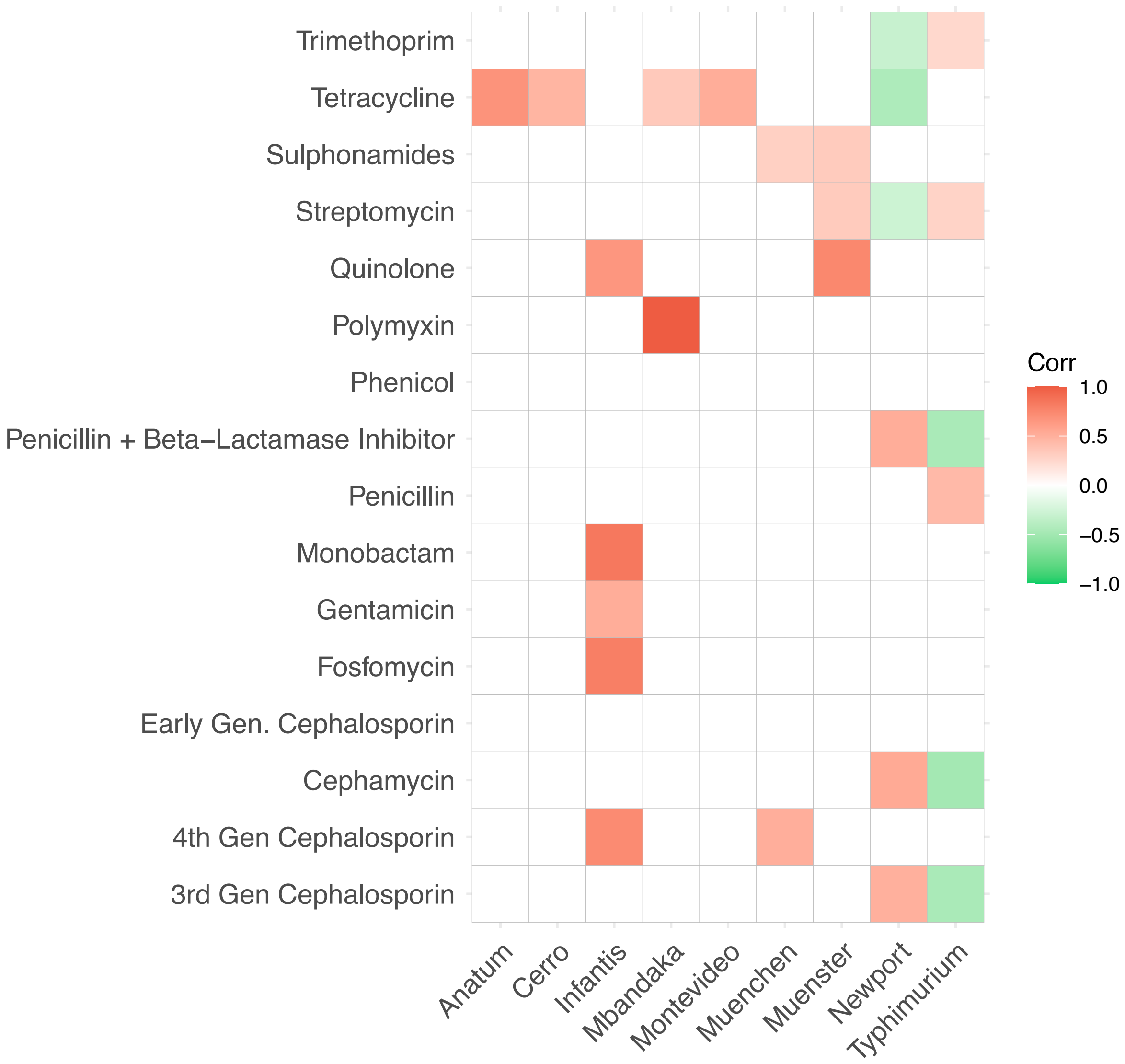

Figure S5. Correlation plot between the most frequent serovars in bovine and resistance phenotypes. Only correlations with an adjusted *p* value below 0.05 are shown. Non-significant correlations are displayed as blank squares.

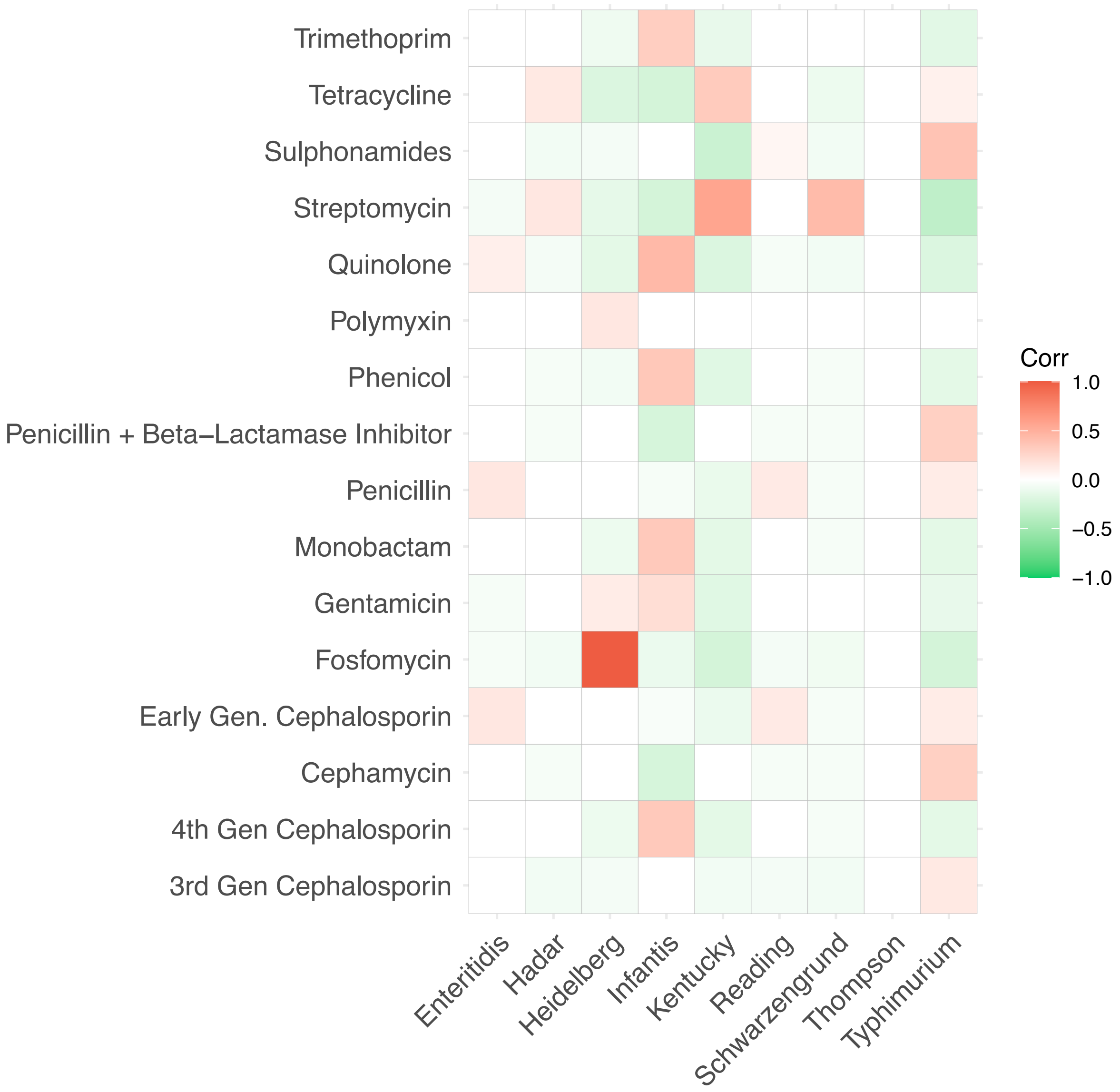

Figure S6. Correlation plot between the most frequent serovars in poultry and resistance phenotypes. Only correlations with an adjusted *p* value below 0.05 are shown. Non-significant correlations are displayed as blank squares.

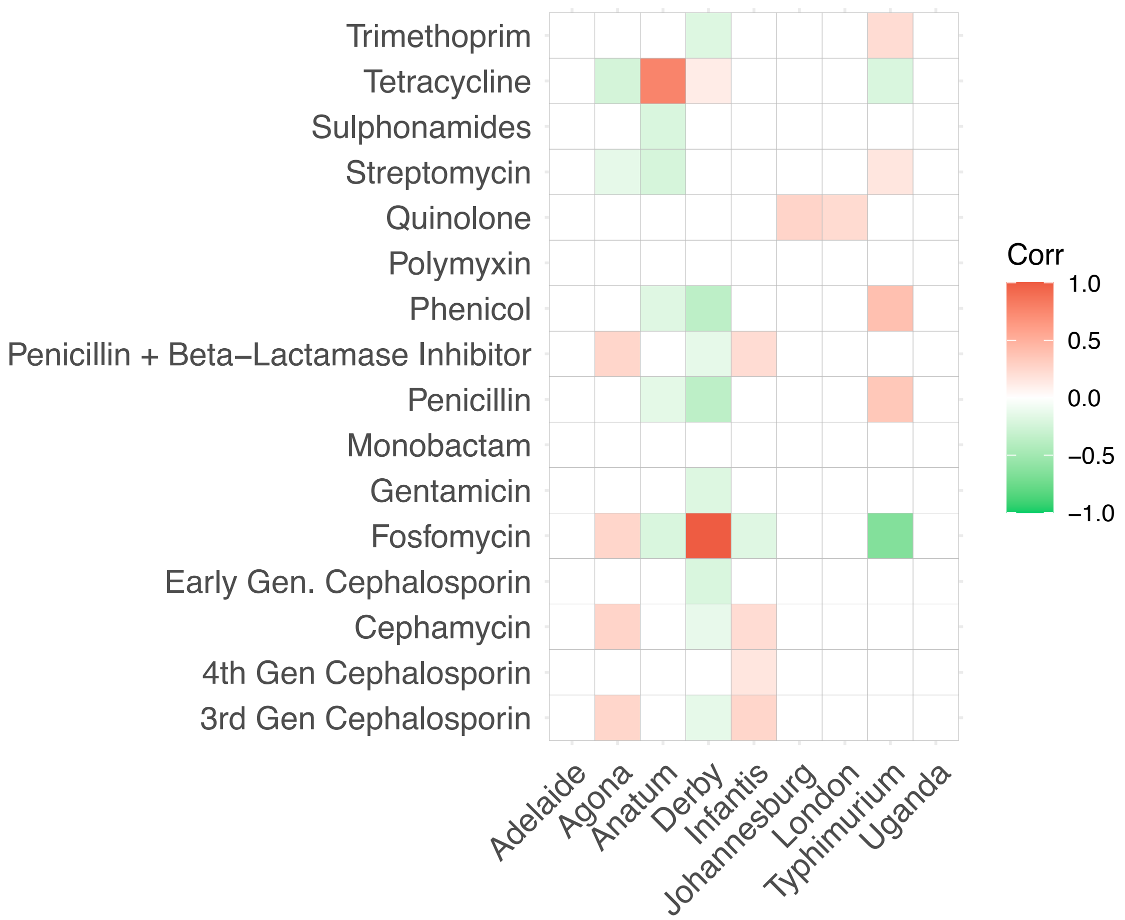

Figure S7. Correlation plot between the most frequent serovars in swine and resistance phenotypes. Only correlations with an adjusted *p* value below 0.05 are shown. Non-significant correlations are displayed as blank squares.

III. Legends for Appendix Tables

Table S1. Metadata file for the 22,102 genomes included for the dataset. Assembly_ID – assembly identifier from the original database; Database – database from which assembly was retrieved; Collection_Date – isolation date; Year – isolation year; Country - isolation country; ISO3 – three letter country code; Latitude – latitude geographic coordinate; Longitude – longitude geographic coordinate; Generic_Host – food-animal host; Source_Niche – indicates wether samples derive from food or from the animal itself; Source_Type – specific animal species; Source_Details – details as available in the database of origin; ST – *Salmonella* Sequence Type; Serovar – *Salmonella* Serovar; BioSample – National Center for Biotechnology Information BioSample accession number; Number_Contigs – number of contigs in assembly; BioProject – National Center for Biotechnology Information BioProject accession number; MDR_Score – calculated Multidrug Resistance Score; Acq_Resist – whether assembly contains acquired resistance gene or not.

Table S2. Output from ResFinder. File_Name – assembly name; Contig – contig name; Start – start position in the contig of the gene identified; End – end position in the contig of the gene identified; Gene – antimicrobial resistance gene identified; Coverage – proportion of gene present in the sequence; Coverage_Map – visual representation of alignment of our sequence against the reference; Gaps – gaps in the sequence versus the reference; Perc_Coverage – proportion of the gene covered; Perc_Identity – proportion of nucleotide matches against reference; Database – reference database; Accession - National Center for Biotechnology Information accession number; Product – gene product; Class – predicted resistance to antimicrobial classes; Phenotype – predicted resistance to individual antimicrobials;

Mechanism of resistance – ResFinder specification of mechanism of resistance if available; Notes – further notes provided by the ResFinder on specific genes; Required_gene – genes required to cause resistance phenotype if any. Gene_clean – Harmonized gene name.

Table S2 can be found in the Zenodo repository in the following link: https://zenodo.org/record/5519129#.YUzEj21Bw4g

Table S3. Output from staramr PointFinder module. Assembly_ID - assembly identifier from the original database, Gene – gene identified with mutation. Mutation designation in brackets; Type – mutation type; Position – amino acid position where mutation occurred; Mutation – specific mutation; Perc_Identity – proportion of nucleotide matches against reference; Perc_Overlap – proportion of the overlap between query and reference; HSP Length/Total Length – high scoring pair length over the length of the gene; Contig – contig name; Start – start position in contig; End – end position in contig.

Table S4. Fitted MDR Score values for all years and hosts. Year – isolation year; Generic_host – animal host; mdr_score – fitted MDR Score; se.fit - standard error; upp_95 – upper bound of 95% confidence interval; low_95 – lower bound of 95% confidence interval.

Table S5. Fitted antimicrobial resistance prevalence for individual classes for all years, hosts. Year - isolation year; Generic_Host – animal host; Phenotype - antimicrobial class; Prevalence – fitted prevalence; low_CI – lower bound of 95% confidence interval; upp_CI – upper bound of 95% confidence interval. signif – wether covariate “Year” was statistically significant or not;

Table S6. Fitted antimicrobial resistance genes' prevalence for all years, hosts. Year – isolation year; Generic_Host – animal host; Gene_Dummy – acronym used to identify antimicrobial resistance gene; Prevalence – fitted prevalence; se.fit – standard error; Gene_clean – antimicrobial resistance gene.

Table S7. Fitted serovar prevalence for all years, hosts. Year – isolation year; Generic_Host – animal host; Serovar – *Salmonella* serovar; Prevalence – fitted prevalence; se.fit – standard error.

Table S8. Fitted serovar prevalence for 2018 and hosts. Year – isolation year; Generic_Host – animal host; Serovar – *Salmonella* serovar; Prevalence – fitted prevalence; se.fit – standard error.

IV. Legend for Dataset S1

Dataset S1. PCU data for all countries between 1985 and 2018. Each column corresponds to a food-animal/year combination. "Ca" refers to bovine, "Ch" refers to poultry, and "Pg" refers to swine.
